# Early left heart decompression protects the lungs in a canine model of acute left heart failure being treated with venoarterial extracorporeal membrane oxygenation

**DOI:** 10.3389/fcvm.2025.1545903

**Published:** 2025-09-24

**Authors:** Yali Chen, Tiewei Xu, Qin Zhen, Changping Gan, Yan Kang, Peng Ji

**Affiliations:** ^1^ Department of Anesthesiology, West China Hospital of Sichuan University, Chengdu, China; ^2^ Laboratory of Anesthesia and Critical Care Medicine, National-Local Joint Engineering Research Centre of Translational Medicine of Anesthesiology, West China Hospital of Sichuan University, Chengdu, China; ^3^ Department of Cardiovascular Surgery, The Third People’s Hospital of Chengdu, Chengdu, China; ^4^ Department of Critical Care Medicine, West China Hospital of Sichuan University, Chengdu, China

**Keywords:** VA-ECMO, left heart decompression, pulmonary function, TNF-α, IL-6

## Abstract

**Background:**

Nearly 30% of patients who undergo venoarterial extracorporeal membrane oxygenation (VA-ECMO) suffer pulmonary edema, which increases mortality risk. Left heart decompression is widely considered an effective way to counter left ventricular dilatation during VA-ECMO, but whether decompression can protect the lung or improve prognosis is unclear. We investigated this question using a canine model of acute left heart failure being treated through VA-ECMO.

**Methods:**

The left anterior descending artery was ligated in 12 beagles to induce acute heart failure, and starting 1 h later, animals were treated using femoral-femoral VA-ECMO for 3 h. In half the animals, left heart decompression was initiated concurrently with VA-ECMO. In the other half, decompression was initiated 1 h after VA-ECMO began. The “early decompression” and “late decompression” groups were compared in terms of pulmonary function, cardiac function, hemodynamics, histopathology and inflammatory responses.

**Results:**

Early initiation of decompression led to significantly higher PaO_2_ (63.27 ± 3.35 vs. 24.70 ± 4.44 mmHg, *P* = 0.030), lower PaCO_2_ (31.65 ± 2.87 vs. 41.02 ± 4.88 mmHg, *P* = 0.014), smaller alveolar-arterial oxygen pressure difference, weaker transpulmonary pressure gradient (3.67 ± 3.14 vs. 13.35 ± 4.26 mmHg, *P* = 0.017), milder pulmonary edema, lower levels of pro-inflammatory cytokines TNF-α and IL-6 in lungs, lower left atrial pressure, lower left ventricular end diastolic pressure, lower mean pulmonary artery pressure, and higher mean arterial pressure. Earlier decompression also led to milder pulmonary blood congestion and pulmonary histopathology.

**Conclusion:**

Left heart decompression, when initiated as soon as possible during VA-ECMO, can protect pulmonary function by alleviating inflammatory responses in the lung, improving hemodynamics and lowering ventricular filling pressure.

## Introduction

Acute heart failure is a leading cause of hospitalization among patients with cardiovascular disease and the most frequent reason for unplanned hospital admissions in patients older than 65 years (1, 2). Treatment outcomes for acute heart failure remain unsatisfactory. Nearly one quarter of affected individuals are re-admitted within 30 days (3), and up to one third of individuals die within 1 year (1). Cardiogenic shock is a low-cardiac-output state, which is the most serious manifestation of acute heart failure (4–6).

While timely diagnosis and treatment of cardiogenic shock can improve prognosis after acute heart failure (7), some patients may require venoarterial extracorporeal membrane oxygenation (VA-ECMO), considered a “last resort” treatment (8–10). While such oxygenation can be effective, 40%–75% of patients die soon after it (11, 12). This is partly because VA-ECMO does not help the left ventricle recover function, it can cause blood stasis within the left ventricle, and it can induce pulmonary edema (13). In fact, at least 20%–30% of patients suffer pulmonary edema after VA-ECMO (14), which increases mortality risk (15). Up to 40% of patients experience pulmonary hemorrhage, which also increases mortality risk (14). Therefore, investigators need to find ways to improve cardiac and pulmonary function under VA-ECMO while minimizing pulmonary edema.

One approach may be left heart decompression, which is currently used in many instances of VA-ECMO (16), yet whose efficacy, indications and optimal timing during VA-ECMO have yet to be established in randomized controlled trials because of ethical concerns. Currently, clinicians resort to left heart decompression as a “remedial” measure only after patients manifest obvious hemodynamic abnormalities. Could “preventive” decompression improve prognosis even in the absence of such abnormalities? If so, is decompression more effective when initiated simultaneously with VA-ECMO or after it has been proceeding for some time?

Studies suggest that the increase in left ventricular load during VA-ECMO increases stress on the ventricular wall, activating mechanical conduction in the heart and release of such pro-inflammatory factors as tumor necrosis factor (TNF)-α and interleukin (IL)-6 (17). The resulting inflammatory processes may cause myocardial fibrosis, ventricular remodeling and cardiomyopathy (17). Given this, could a “preventive” LHD strategy achieve cardiopulmonary protection by mitigating inflammatory responses?

We addressed these questions in a canine model of acute heart failure treated by VA-ECMO. We examined whether initiating left heart decompression at the same time as VA-ECMO or well after onset of VA-ECMO improved cardiac and pulmonary outcomes and inflammatory responses. We hypothesized that left heart decompression improves pulmonary and cardiac outcomes at least in part by dampening inflammatory responses.

## Methods

### Canine model of acute heart failure

Animal procedures were approved by the Animal Ethics Committee of West China Hospital of Sichuan University (approval 2019107A) and conducted with ARRIVE guidelines. Adult male beagle dogs 1–2 years old and weighing 10–11 kg were purchased from Chengdu Dassy Biological Technology (Chengdu, China) and housed individually at 22°C–24°C and relative humidity 45%–55%, with lights on from 7:00 to 19:00. Animals had *ad libitum* access to chow and water.

After midnight on the night before surgery, dogs were not provided solid food or water. Half an hour before surgery, atropine (0.02 mg/kg) was injected intramuscularly to reduce secretion in the mouth and trachea and to prevent airway obstruction. A venous indwelling needle (20 G) was inserted into the median elbow vein of the dog's right upper limb, and anesthesia was induced using 0.1 mg/kg midazolam, 0.3 μg/kg sufentanil, 3–4 mg/kg propofol, and 0.1 mg/kg vecuronium bromide. After laryngeal reflexes were confirmed to be adequately suppressed, an endotracheal tube of inner diameter 7.0 mm with cuff was inserted to a depth of 25–26 cm from the incisors. Mechanical ventilation was initiated at a tidal volume of 10–15 ml/kg and a respiration rate of 20 beats/min in order to maintain end tidal CO_2_ partial pressure (P_ET_CO_2_) of 35–45 mmHg. Anesthesia was maintained using propofol, midazolam, sufentanil and vecuronium bromide. Body termperature was maintained at 37.5°C–38.5°C using thermal blankets.

During surgery, dogs were monitored in terms of electrocardiographic activity, blood oxygen saturation (pulse oximetry), temperature, P_ET_CO_2_ and airway pressure. The middle and lower one-third of the left anterior descending coronary artery was ligated as described (18) to induce left cardiac dysfunction due to myocardial ischemia. Acute heart failure was defined to be achieved if echocardiography showed obviously abnormal segmental motion of the left ventricle and ST-segment elevation, if the ejection fraction (EF) was less than 35% during sinus rhythm, and if left atrial pressure (LAP) exceeded 15 mmHg (19).

### Peripheral VA-ECMO

After 1 h of coronary occlusion, heparin was administered at 300 U/kg and the activated clotting time was monitored for longer than 400 s. A femoral venous catheter (14 Fr; Mindray, Shenzhen, China) was inserted into the right femoral vein, and a femoral artery catheter (8 Fr; Mindray) was inserted through the right femoral artery. The two femoral catheters were connected to a VA-ECMO circuit (Maquet Cardiopulmonary, Rastatt, Germany) that had been filled with 150 ml Ringer's lactate solution, 200 ml 6% hydroxyethyl starch and 20 mg heparin. The circuit was operated with the following parameters: flow rate, 100 ml/kg/min; ratio of gas flow to blood flow, 1:1; and oxygen concentration, 40%. Ventilation was performed at the following parameters: respiratory rate, 10 breaths/min; tidal volume, 10–15 ml/kg; ratio of inspiration to expiration, 1:2; peak inspiratory pressure, 10 cmH_2_O; and oxygen concentration, 21%. VA-ECMO was conducted for a total of 3 h.

Throughout VA-ECMO, lidocaine was administered continuously at 1.5 mg/kg/h to prevent ventricular arrhythmia. If ventricular fibrillation occurred, animals were defibrillated using the HEARTSTART XL system (Philips, Amsterdam, Netherlands) and given 3 mg/kg amiodarone.

### Left heart decompression

Animals were randomized to receive left heart decompression for 3 h beginning in tandem with VA-ECMO (hereafter “early decompression”) or for 2 h beginning after 1 h of VA-ECMO (hereafter “late decompression”). For decompression, a DLP® arterial catheter (6 Fr; Medtronic, Minneapolis, MN, USA) was inserted under ultrasound guidance to a depth of 1.5 cm through the apex of the left ventricle. The catheter was positioned to avoid obstructing the outflow tract of the left ventricle or the mitral valve and to avoid coming too close to the ventricular wall. The decompression tube was connected to a centrifugal pump and to the venous end of the VA-ECMO circuit, in which the flow rate was 40 ml/kg/min.

We did not include a control group of animals that did not receive left heart decompression because in preliminary experiments, few such animals survived until the end of surgery due to extremely high LAP (data not shown).

### Evaluation of pulmonary function

Pulmonary function was assessed in terms of oxygen partial pressure (PaO_2_), carbon dioxide partial pressure (PaCO_2_), the alveolar-arterial oxygen pressure difference (A-aDO_2_), and transpulmonary pressure gradient (TPG). These parameters were measured at the following time points: baseline (T0), 1 h after coronary artery ligation (T1), after 1 h of VA-ECMO (T2) and after 3 h of VA-ECMO (T3). At each time point, blood (0.5 ml) from the left ventricle was analyzed using an i-STAT blood gas analyzer (Abbott Laboratories, Chicago, IL, USA).

A-aDO_2_ was calculated using the formula A−aDO_2_ = PAO_2_−PaO_2_, where PAO_2_ = [FiO_2_ × (P_atm_−P_H2O_)]−(PaCO_2_/R). TPG was calculated using the formula TPG = PAP−LAP, where PAP refers to pulmonary artery pressure and LAP to left atrial pressure. These parameters were measured as described below in “Evaluation of hemodynamics”.

### Evaluation of cardiac function

Cardiac function was assessed using two-dimensional epicardial echocardiography because the narrow, elongated thorax of dogs leads to poor imaging quality with transthoracic ultrasonography. At a probe frequency of 10 MHz and image depth of 6–10 cm, the following views were recorded during three consecutive cardiac cycles: apical four-chamber, papillary muscle short-axis, and left ventricular long-axis. The following parameters were averaged over the three consecutive cardiac cycles: left atrial (LA), left ventricular (LV), EF, CO, tricuspid annular plane systolic excursion (TAPSE) and left ventricular stroke work index (LVSWI).

### Evaluation of hemodynamics

Animals were anesthetized, an area of skin on the anterior median chest was disinfected, and an incision was made, through which the chest and pericardium were opened. A Swan Ganz Thermodilution catheter (5 Fr; Edwards Lifesciences, Irvine, CA, USA) containing heparin solution was inserted into the left atrium, aortic root, pulmonary artery and left ventricle. This catheter was used to measure heart rate, systolic and diastolic pressure at the aortic root, LAP, left ventricular end diastolic pressure (LVEDP), and mean pulmonary artery pressure. The signals were recorded using a BL-420S signal acquisition system (Taimeng, Chengdu, China). All pressure transducers were zeroed to atmospheric pressure and leveled at the dog's manubrium. Coronary perfusion pressure (CPP) and mean blood pressure (MBP) at the aortic root were calculated according to the corresponding formulas.

At the same time points, blood from the LV and pulmonary artery was sampled and assayed for lactic acid and pulmonary arterial oxygen saturation (SmO_2_).

### Evaluation of histopathology and pro-inflammatory cytokines

At the end of the experiment, biopsies (0.5 cm^3^) were taken from the inferior lobe of the left lung, apex of the left ventricle and right ventricular outflow tract; the biopsies were sectioned, stained with hematoxylin-eosin, and assessed for lung and myocardial histopathology as described (20–22). In parallel, a replicate set of biopsies from these tissues as well as plasma from blood was assayed for IL-6 and TNF-α using commercial kits (catalog nos. 11802 and 11943, Meimian, Yancheng, China). Assay samples were analyzed in triplicate and the results were averaged.

Immediately after VA-ECMO, the left lungs of dogs were removed, weighed while wet, then dried in an oven at 60°C–70°C until constant weight; the ratio of wet to dry weight served as an indicator of pulmonary edema (23).

### Statistical analysis

Data were analyzed statistically using SPSS 15 (IBM, Chicago, IL, US). All data were confirmed to show normal distributions based on the Kolmogorov Smirnov test as well as homogeneous variance based on the Brown Forsythe test. Differences between the early and late decompression groups were assessed for significance using the independent-samples *t* test, while intragroup differences between measurements at different time points were assessed for significance using two-way analysis of variance for repeated measures, followed by a Bonferroni *post hoc* test.

## Results

**Figure 1:**
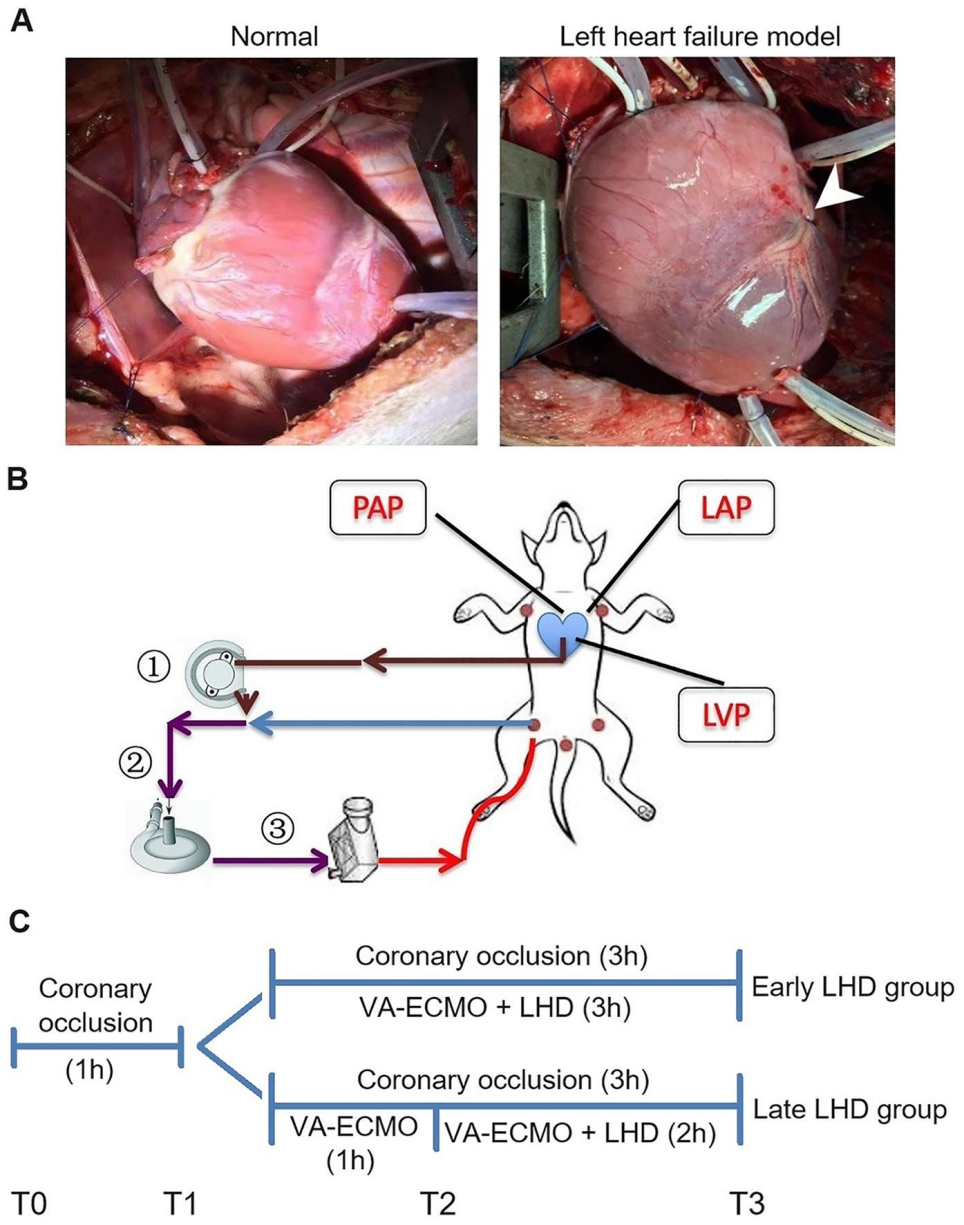
Establishment of the AHF model and peripheral VA-ECMO with LHD model in dogs. **(A)** Left: The heart of a normal dog. Right: The heart of a dog after ligating the anterior descending branch of the coronary artery. **(B)** The schematic diagram of the VA-ECMO with LHD model. **(C)** The experimental timeline. AHF, acute heart failure; LAP, left atrial pressure; LHD, left heart decompression; LVP, left ventricle pressure; PAP, pulmonary artery pressure; VA-ECMO, venoarterial extracorporeal membrane oxygenation.

In preliminary experiments, six beagle dogs weighing 10.35 ± 0.47 kg were subjected to ligation of the left anterior descending coronary artery, which showed that acute heart failure occurred by 1 h afterward (Figure 1A). Therefore VA-ECMO in the main experiments was initiated at this time point. In addition, we found that ventricular fibrillation was clinically significant after 1 h of VA-ECMO, so that was the time point at which we initiated left heart decompression in the “late decompression” group (Figure 1B). Finally, the preliminary studies showed that a decompression flow rate of 40 ml/kg/min was superior to 20 or 60 ml/kg/min (Supplementary Figure S1), so this rate was used in the main experiments.

**Figure 2:**
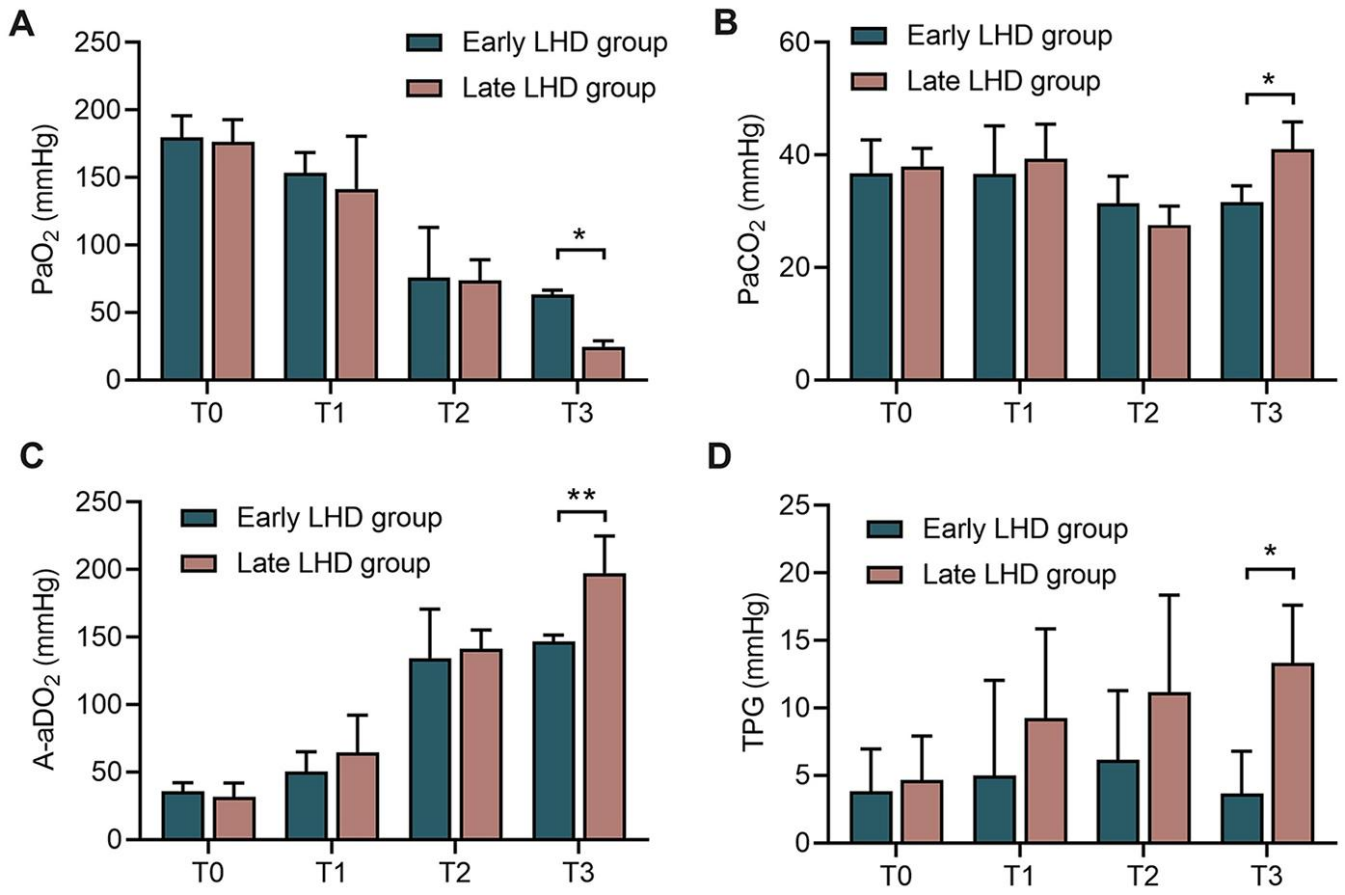
The changes of pulmonary function in dogs at different time between early LHD group and late LHD group. The change of PaO_2_ **(A)**, PaCO_2_ **(B)**, A-aDO_2_ **(C)**, TPG **(D)** in dogs at different time between early LHD group and late LHD group. A-aDO_2_, alveolar-arterial oxygen pressure difference; LHD, left heart decompression; PaO_2_, oxygen partial pressure; PaCO_2_, carbon dioxide pressure; TPG, transpulmonary pressure gradient. Data were present as mean ± SD, and were compared by two-way ANOVA. *, *P* < 0.05; **, *P* < 0.01.

The main experiments involved 12 beagle dogs randomized 1:1 into an early decompression group (body weight, 10.50 ± 0.77 kg) and late decompression group (body weight, 10.70 ± 1.06 kg) (Figure 1C). At the end of the experiment, early decompression protected pulmonary function significantly better than late decompression as reflected in higher PaO_2_ (63.27 ± 3.35 vs. 24.70 ± 4.44 mmHg, *P* = 0.030; Figure 2A), lower PaCO_2_ (31.65 ± 2.87 vs. 41.02 ± 4.88 mmHg, *P* = 0.014; Figure 2B), lower A-aDO_2_ (146.72 ± 4.87 vs. 197.35 ± 27.51 mmHg, *P* = 0.003; Figure 2C), and lower TPG (3.67 ± 3.14 vs. 13.35 ± 4.26 mmHg, *P* = 0.017; Figure 2D).

**Figure 3:**
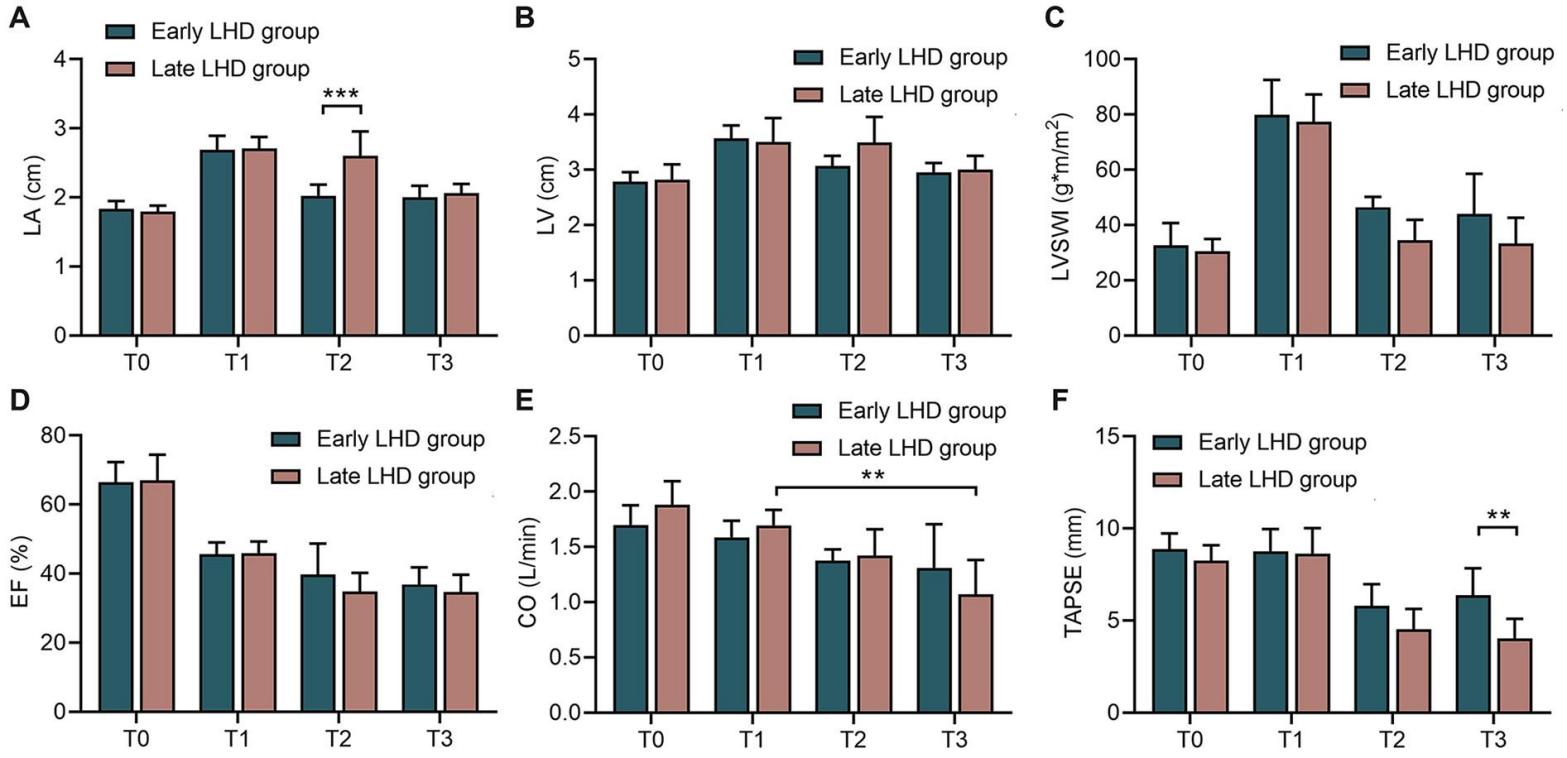
The changes of echocardiographic indicators in dogs at different time between early LHD group and late LHD group. The changes of LA **(A)**, LV **(B)**, LVSWI **(C)**, EF **(D)**, CO **(E)** and TAPSE **(F)** at different time between early LHD group and late LHD group. CO, cardiac output; EF, ejection fraction; LA, left atrial; LHD, left heart decompression; LV, left ventricle; LVSWI, left ventricular stroke work index; TAPSE, tricuspid annular plane systolic excursion. Data were present as mean ± SD, and were compared by two-way ANOVA. *, *P* < 0.05; **, *P* < 0.01; ***, *P* < 0.001.

Early decompression also protected cardiac function better than late decompression, at least according to some parameters. Early decompression led to significantly smaller LA at 1 h after VA-ECMO (2.03 ± 0.16 vs. 2.61 ± 0.35 cm, *P* < 0.001; Figure 3A), although this difference disappeared by the end of the experiment; and it led to significantly higher TAPSE at the end of the experiment (6.38 ± 1.46 vs. 4.03 ± 1.06 mm, *P* = 0.006, Figure 3F). In the early LHD group, there was no statistical difference in CO between T1 and T3. However, in the late LHD group, CO at T3 was significantly lower than that at T1 (1.069 ± 0.314 vs. 1.695 ± 0.140 L/min, *P* < 0.001; Figure 3E), indicating that early LHD has a protective effect on CO. Nevertheless, timing of decompression did not significantly affect LV (Figure 3B), LVSWI (Figure 3C), EF (Figure 3D) or CO (Figure 3E) at any time point.

**Figure 4:**
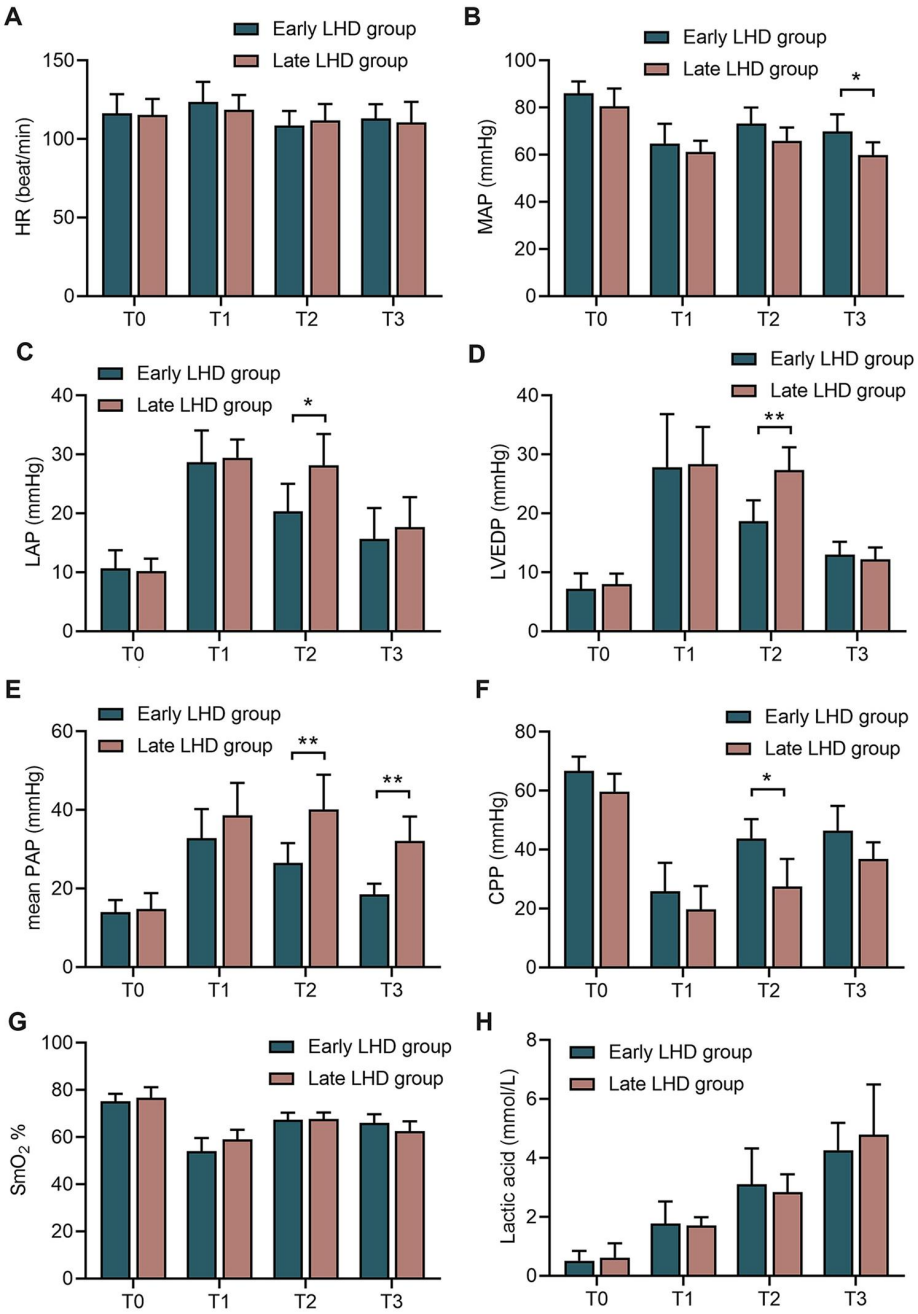
The changes of circulatory indexes in dogs at different time between early LHD group and late LHD group. The change of HR **(A)**, MAP **(B)**, LAP **(C)**, LVEDP **(D)**, mean PAP **(E)**, CPP **(F)**, SmO_2_ **(G)**, lactic acid **(H)** and in dogs at different time between early LHD group and late LHD group. CPP, coronary perfusion pressure; HR, heart rate; LAP, left atrial pressure; LHD, left heart decompression; LVEDP, left ventricular end diastolic pressure; MAP, mean arterial blood pressure; PAP, pulmonary artery pressure; SmO_2_, pulmonary arterial oxygen saturation. Data were present as mean ± SD, and were compared by two-way ANOVA. *, *P* < 0.05; **, *P* < 0.01; ***, *P* < 0.001.

Early decompression led to better hemodynamics than late decompression in terms of significantly lower LAP (20.33 ± 4.68 vs. 28.17 ± 5.31 mmHg, *P* = 0.017; Figure 4C), lower LVEDP (18.67 ± 3.56 vs. 27.33 ± 3.88 mmHg, *P* = 0.009; Figure 4D), lower mean PAP (26.50 ± 5.09 vs. 40.17 ± 8.80 mmHg vs., *P* = 0.002; Figure 4E), and higher CPP (43.83 ± 6.52 vs. 27.50 ± 9.35 mmHg, *P* = 0.015, Figure 4F) at 1 h after VA-ECMO. The benefits of early compression remained visible at the end of the experiment in terms of higher MBP (69.35 ± 17.91 vs. 59.95 ± 15.39 mmHg, *P* = 0.047; Figure 4B) and lower mean PAP (32.17 ± 6.18 vs. 18.50 ± 2.74 mmHg, *P* = 0.002; Figure 4E).

Timing of left heart decompression did not significantly affect the ability of VA-ECMO to perfuse the body: the early and late groups did not differ significantly in SmO_2_ (Figure 4F) or lactic acid (Figure 4G) at any time point.

**Figure 5:**
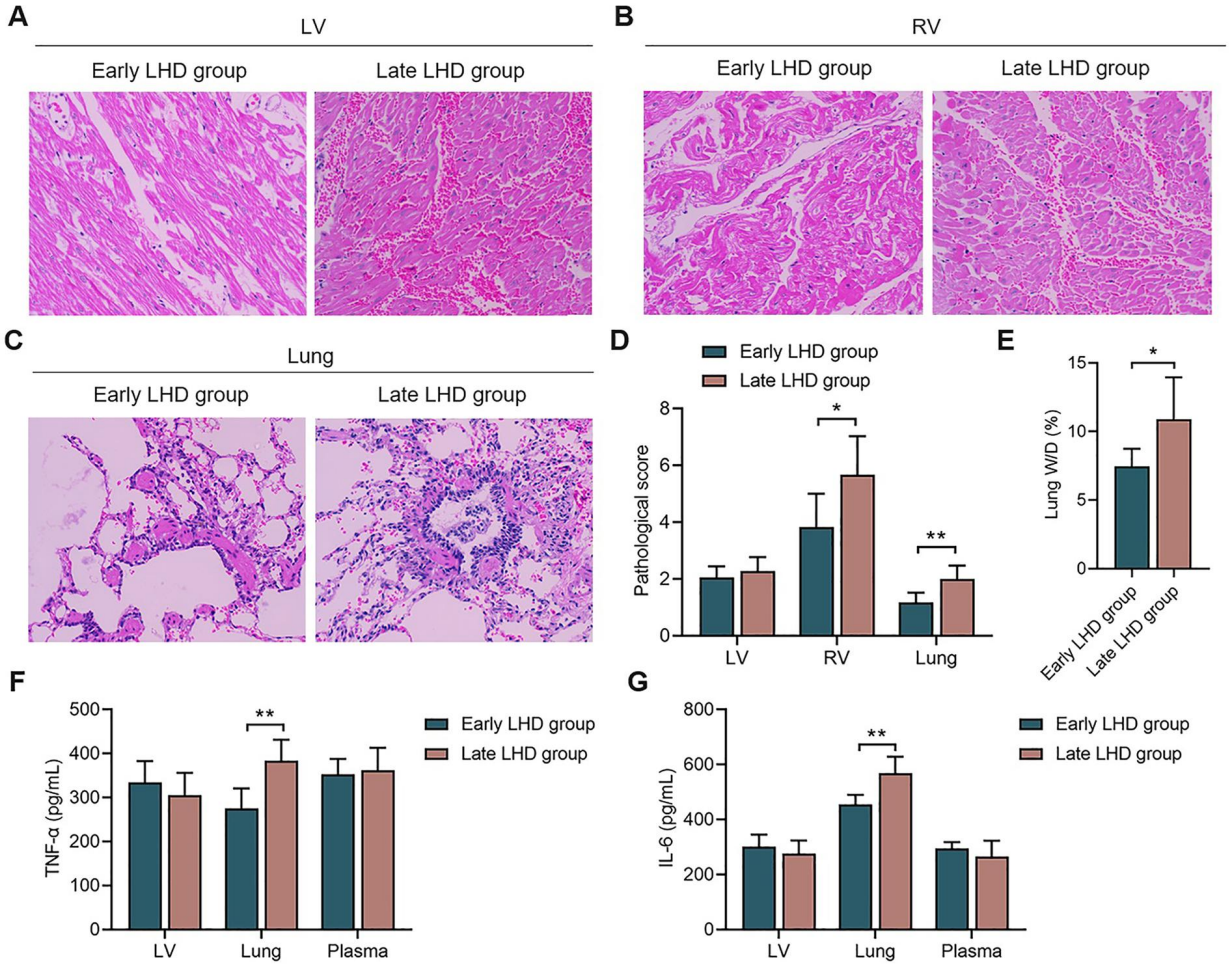
The histopathological changes and inflammatory reaction of myocardium and lung. The histopathological changes of LV myocardium **(A)**, RV myocardium **(B)**, and lung **(C)** between early LHD group and late LHD group. **(D)** The pathological score of LV myocardium, RV myocardium, and lung between early LHD group and late LHD group. **(E)** The lung W/D ratio between early LHD group and late LHD group. The concentrations of TNF-α **(F)** and IL-6 **(G)** in myocardium, lung and plasma between early LHD group and late LHD group. IL-6, interleukin-6; LV, left ventricular; LHD, left heart decompression; RV, right ventricular; TNF-α, tumor necrosis factor-α. Data were present as mean ± SD, and were compared by *t*-test. *, *P* < 0.05; **, *P* < 0.01.

Myocardium and lung showed edema, hemorrhage and infiltration by inflammatory cells regardless of whether decompression was initiated early or late (Figures 5A–C), but early decompression was associated with significantly less hemorrhaging and inflammatory cell infiltration, whether in the right ventricular myocardium or lung (Figure 5D). On the other hand, histopathology of the left ventricular myocardium was similar between the early and late groups.

Early decompression was associated with a significantly lower ratio of wet to dry weight of the left lung at the end of the experiment (7.46 ± 1.28% vs. 10.88 ± 3.06%, *P* = 0.031; Figure 5E), suggesting milder pulmonary edema.

While timing of decompression did not significantly affect levels of TNF-α and IL-6 in myocardium or plasma, early decompression was associated with significantly lower levels of TNF-α (275.62 ± 44.95 vs. 383.92 ± 47.63 pg/ml, *P* = 0.002; Figure 5F) and IL-6 (454.68 ± 34.71 vs. 568.65 ± 59.84 pg/ml, *P* = 0.006; Figure 5G) in the lung. These results link more severe lung injury to stronger inflammatory responses, and they support our hypothesis that the ability of decompression to protect lung function involves attenuation of inflammatory processes.

## Discussion

Our experiments in a canine model of acute heart failure being treated with VA-ECMO is that left heart decompression, when initiated simultaneously with VA-ECMO, can significantly mitigate the procedure's harmful effects on pulmonary and cardiac function. These protective effects are associated with mitigation of inflammatory responses and improvement in hemodynamics, reducing left heart filling pressure. Our findings justify clinical studies to develop and optimize left heart decompression as an approach to alleviate or even prevent the lung and heart injury that VA-ECMO often causes, but also to improve recovery of cardiac function.

In our model, as in other animal models and patients, VA-ECMO induced lung edema, hyaline membranes, alveolar hemorrhage, thrombosis and focal necrosis (24–26). VA-ECMO imposes an afterload on the failing left ventricle, which can prevent the aortic valve from opening normally and thereby induce blood stasis in that ventricle (27). In parallel, numerous factors can increase preload on the left ventricle, dilating it and elevating LVEDP. The myocardium consumes increasing amounts of oxygen as coronary perfusion falls, prolonging myocardial ischemia. The persistently elevated LVEDP impairs pulmonary venous return, which can cause pulmonary edema and even induce secondary pulmonary hypertension, compromising right heart function. Indeed, left heart decompression has been shown to alleviate pulmonary edema and improve pulmonary dysfunction in patients undergoing cardiopulmonary bypass surgery (28) and to rapidly alleviate pulmonary edema and pulmonary hemorrhage in patients receiving VA-ECMO (29).

Using a canine model allowed us to compare the efficacy of early or decompression, and our results strongly indicate that early decompression is superior. Early decompression may be much better than late decompression because it can prevent the onset of cardiogenic pulmonary edema. Such edema impairs pulmonary diffusion capacity, reduces local alveolar oxygen partial pressure, and disrupts alveolar epithelial function (6). Alveolar hypoxia hinders pulmonary fluid clearance and the production of surfactant, leading to the accumulation of proteins, lactate dehydrogenase, neutrophils, and elastase in alveoli, inducing localized and systemic inflammation (30, 31). Contact of the blood with the extracorporeal membrane can also trigger systemic inflammatory responses mediated by TNF-α and by IL-6, IL-8 and IL-10 (32). While early decompression in our canine model led to significantly lower levels of TNF-α and IL-6, milder histopathology and less neutrophil infiltration in the bronchi and alveoli than late decompression did, the two methods led to similar intensity of systemic and myocardial inflammatory responses. This suggests that the observed ability of early left heart decompression to protect the lung during VA-ECMO does not involve mitigation of systemic inflammation, but instead direct mitigation of pulmonary inflammation. We speculate that early decompression improves pulmonary oxygenation and alleviates pulmonary edema, thereby interrupting the vicious cycle of alveolar hypoxia and inflammation.

Our results support the idea of adding left heart decompression to the list of published methods to prevent left ventricular distension during VA-ECMO, which already includes adjusting the flow rate, applying lung-protective ventilation, administering diuretics and renal replacement therapy to avoid volume overload, or administering inotropic drugs and vasodilators to improve CO and reduce pulmonary pressure (33, 34). Future studies should compare the effects of these various approaches on the prognosis of different types of patients undergoing VA-ECMO.

The optimal timing for left heart decompression during VA-ECMO remains heterogeneous in clinical practice. Some studies suggest that a visibly enlarged left ventricle on echocardiography may serve as an indication for decompression (35, 36). However, this approach has significant limitations. In pathophysiological states, the relationship between LV size and LVEDP is not linear, largely due to alterations in intrinsic myocardial compliance and interindividual variability in baseline cardiac anatomy and function (37, 38). Moreover, pericardial constraint may further obscure the hemodynamic interpretation of LV dimensions. Consequently, relying solely on echocardiographic assessment of LV size to guide decompression may be insufficient to accurately identify patients experiencing significant LV distension and elevated filling pressures.

While LVSWI, EF and CO are valuable parameters for assessing LV performance, its accurate quantification by echocardiography is subject to multiple limitations, particularly in the setting of VA-ECMO and surgical interventions such as LV venting. Factors such as preload and afterload variability, continuous flow from the ECMO circuit, interference from the decompression cannula, as well as restricted acoustic windows can significantly affect pressure and volume estimations needed for them calculation. Given these technical challenges and the potential for inconsistent measurements across time points, we decided not to perform within-group comparisons of them. This approach was taken to avoid overinterpretation of potentially unreliable data in this dynamic and highly invasive setting. Similar concerns regarding the limitations of echocardiographic-derived them have been raised in prior studies (39–41).

In this study, left ventricular decompression was achieved via surgical placement of a venting catheter through apical puncture. This approach was selected for its technical feasibility in the animal model; however, it may result in myocardial injury and impaired ventricular function, and is more commonly used in clinical settings for bridge-to-durable ventricular assist device (VAD) implantation rather than routine decompression. Percutaneous microaxial transaortic ventricular assist devices (pVADs) are emerging as a less invasive and more clinically relevant option for LV unloading during VA-ECMO. Future studies are planned to evaluate the timing, hemodynamic impact, and pulmonary protective effects of pVAD-mediated decompression in a similar model.

Our results should be interpreted with caution given that, for ethical reasons, our study lacked a control group that did not receive left heart decompression. Further preclinical studies should determine whether the observed lack of influence of early or late decompression on myocardial or systemic inflammatory responses depends on the severity of myocardial injury or length of experimental time.

Despite these limitations, our study of a canine model provides strong evidence that early, but not late, left heart decompression can reduce LVEDP, improve lung oxygenation, and mitigate lung injury during VA-ECMO after acute heart failure. Decompression may exert these effects by reducing pulmonary congestion and local inflammatory responses.

## Data Availability

The original contributions presented in the study are included in the article/Supplementary Material, further inquiries can be directed to the corresponding author.

## References

[1] Emmons-Bell S Johnson C Roth G. Prevalence, incidence and survival of heart failure: a systematic review. Heart. (2022) 108(17):1351–60. 10.1136/heartjnl-2021-320131 35042750 PMC9380485

[2] Deniau B Costanzo MR Sliwa K Asakage A Mullens W Mebazaa A. Acute heart failure: current pharmacological treatment and perspectives. Eur Heart J. (2023) 44(44):4634–49. 10.1093/eurheartj/ehad617 37850661

[3] Arrigo M Jessup M Mullens W Reza N Shah AM Sliwa K Acute heart failure. Nat Rev Dis Primers. (2020) 6(1):16. 10.1038/s41572-020-0151-7 32139695 PMC7714436

[4] Reynolds HR Hochman JS. Cardiogenic shock: current concepts and improving outcomes. Circulation. (2008) 117(5):686–97. 10.1161/CIRCULATIONAHA.106.613596 18250279

[5] van Diepen S Katz JN Albert NM Henry TD Jacobs AK Kapur NK Contemporary management of cardiogenic shock: a scientific statement from the American heart association. Circulation. (2017) 136(16):e232–68. 10.1161/CIR.0000000000000525 28923988

[6] Fröhlich S Boylan J McLoughlin P. Hypoxia-induced inflammation in the lung: a potential therapeutic target in acute lung injury? Am J Respir Cell Mol Biol. (2013) 48(3):271–9. 10.1165/rcmb.2012-0137TR 23087053

[7] Shiraishi Y Kawana M Nakata J Sato N Fukuda K Kohsaka S. Time-sensitive approach in the management of acute heart failure. ESC Heart Failure. (2021) 8(1):204–21. 10.1002/ehf2.13139 33295126 PMC7835610

[8] Hoyler MM Flynn B Iannacone EM Jones MM Ivascu NS. Clinical management of venoarterial extracorporeal membrane oxygenation. J Cardiothorac Vasc Anesth. (2020) 34(10):2776–92. 10.1053/j.jvca.2019.12.047 32139341

[9] Rihal CS Naidu SS Givertz MM Szeto WY Burke JA Kapur NK 2015 SCAI/ACC/HFSA/STS clinical expert consensus statement on the use of percutaneous mechanical circulatory support devices in cardiovascular care: endorsed by the American heart association, the cardiological society of India, and Sociedad Latino Americana de Cardiologia Intervencion; affirmation of value by the Canadian association of interventional cardiology-association Canadienne de Cardiologie d'intervention. J Am Coll Cardiol. (2015) 65(19):e7–26. 10.1016/j.jacc.2015.03.036 25861963

[10] Paden ML Conrad SA Rycus PT Thiagarajan RR. Extracorporeal life support organization registry report 2012. ASAIO J. (2013) 59(3):202–10. 10.1097/MAT.0b013e3182904a52 23644605

[11] Thiagarajan RR Barbaro RP Rycus PT McMullan DM Conrad SA Fortenberry JD Extracorporeal life support organization registry international report 2016. ASAIO J. (2017) 63(1):60–7. 10.1097/MAT.0000000000000475 27984321

[12] Meani P Pappalardo F. The step forward for VA ECMO: left ventricular unloading!. J Thorac Dis. (2017) 9(11):4149–51. 10.21037/jtd.2017.10.14 29268456 PMC5721012

[13] Rao P Khalpey Z Smith R Burkhoff D Kociol RD. Venoarterial extracorporeal membrane oxygenation for cardiogenic shock and cardiac arrest. Circ Heart Fail. (2018) 11(9):e004905. 10.1161/CIRCHEARTFAILURE.118.004905 30354364

[14] Saeed O Nunez JI Jorde UP. Pulmonary protection from left ventricular distension during venoarterial extracorporeal membrane oxygenation: review and management algorithm. Lung. (2023) 201(2):119–34. 10.1007/s00408-023-00616-8 37043003

[15] Distelmaier K Wiedemann D Lampichler K Toth D Galli L Haberl T Interdependence of VA-ECMO output, pulmonary congestion and outcome after cardiac surgery. Eur J Intern Med. (2020) 81:67–70. 10.1016/j.ejim.2020.07.014 32736947

[16] Xie A Forrest P Loforte A. Left ventricular decompression in veno-arterial extracorporeal membrane oxygenation. Ann Cardiothorac Surg. (2019) 8(1):9–18. 10.21037/acs.2018.11.07 30854308 PMC6379183

[17] Hochman JS. Cardiogenic shock complicating acute myocardial infarction: expanding the paradigm. Circulation. (2003) 107(24):2998–3002. 10.1161/01.CIR.0000075927.67673.F2 12821585

[18] Lin J Qin Z Qian H Li Y Luo N Du L. A novel catheter with retractable stent that can prevent aortic insufficiency during left ventricular assist. PLoS One. (2018) 13(4):e0194658. 10.1371/journal.pone.0194658 29608576 PMC5880366

[19] Ishikawa K Aguero J Tilemann L Ladage D Hammoudi N Kawase Y Characterizing preclinical models of ischemic heart failure: differences between LAD and LCx infarctions. Am J Physiol Heart Circ Physiol. (2014) 307(10):H1478–86. 10.1152/ajpheart.00797.2013 25217654 PMC4233300

[20] Matute-Bello G Downey G Moore BB Groshong SD Matthay MA Slutsky AS An official American thoracic society workshop report: features and measurements of experimental acute lung injury in animals. Am J Respir Cell Mol Biol. (2011) 44(5):725–38. 10.1165/rcmb.2009-0210ST 21531958 PMC7328339

[21] Wang W Liang X Fu D Tie R Xing W Ji L Apocynum venetum leaf attenuates myocardial ischemia/reperfusion injury by inhibiting oxidative stress. Am J Chin Med. (2015) 43(1):71–85. 10.1142/S0192415X15500056 25579758

[22] Lu L Xu K Zhang LJ Morelli J Krazinski AW Silverman JR Lung ischaemia-reperfusion injury in a canine model: dual-energy CT findings with pathophysiological correlation. Br J Radiol. (2014) 87(1036):20130716. 10.1259/bjr.20130716 24611753 PMC4067035

[23] Xiong Y Yao H Cheng Y Gong D Liao X Wang R. Effects of monoacylglycerol lipase inhibitor URB602 on lung ischemia-reperfusion injury in mice. Biochem Biophys Res Commun. (2018) 506(3):578–84. 10.1016/j.bbrc.2018.10.098 30366666

[24] Koul B Willen H Sjöberg T Wetterberg T Kugelberg J Steen S. Pulmonary sequelae of prolonged total venoarterial bypass: evaluation with a new experimental model. Ann Thorac Surg. (1991) 51(5):794–9. 10.1016/0003-4975(91)90128-D 2025083

[25] Mizuno T Tatsumi E Nishinaka T Katagiri N Ohikawa M Naito H Observation of alveolar fibrosis in a goat following venoarterial bypass for up to 5 months using extracorporeal membrane oxygenation. J Artif Organs. (2004) 7(2):107–9. 10.1007/s10047-004-0248-x 15309678

[26] Chou P Blei ED Shen-Schwarz S Gonzalez-Crussi F Reynolds M. Pulmonary changes following extracorporeal membrane oxygenation: autopsy study of 23 cases. Hum Pathol. (1993) 24(4):405–12. 10.1016/0046-8177(93)90089-Y 8491481

[27] Truby LK Takeda K Mauro C Yuzefpolskaya M Garan AR Kirtane AJ Incidence and implications of left ventricular distention during venoarterial extracorporeal membrane oxygenation support. ASAIO J. (2017) 63(3):257–65. 10.1097/MAT.0000000000000553 28422817

[28] Ok YJ Jung SH Lee SW Ahn JM Lim JY. Efficacy of left heart decompression during extracorporeal membrane oxygenation: a case-control study. J Thorac Dis. (2019) 11(3):865–72. 10.21037/jtd.2019.01.110 31019775 PMC6462734

[29] Baruteau AE Barnetche T Morin L Jalal Z Boscamp NS Le Bret E Percutaneous balloon atrial septostomy on top of venoarterial extracorporeal membrane oxygenation results in safe and effective left heart decompression. Eur Heart J Acute Cardiovasc Care. (2018) 7(1):70–9. 10.1177/2048872616675485 27742755

[30] Jain M Sznajder JI. Effects of hypoxia on the alveolar epithelium. Proc Am Thorac Soc. (2005) 2(3):202–5. 10.1513/pats.200501-006AC 16222038

[31] Siepe M Goebel U Mecklenburg A Doenst T Benk C Stein P Pulsatile pulmonary perfusion during cardiopulmonary bypass reduces the pulmonary inflammatory response. Ann Thorac Surg. (2008) 86(1):115–22. 10.1016/j.athoracsur.2008.03.062 18573409

[32] Kotani T Kotake Y Morisaki H Takeda J Shimizu H Ueda T Activation of a neutrophil-derived inflammatory response in the airways during cardiopulmonary bypass. Anesth Analg. (2006) 103(6):1394–9. 10.1213/01.ane.0000243391.05091.bb 17122209

[33] Roumy A Liaudet L Rusca M Marcucci C Kirsch M. Pulmonary complications associated with veno-arterial extra-corporeal membrane oxygenation: a comprehensive review. Critical Care. (2020) 24(1):212. 10.1186/s13054-020-02937-z 32393326 PMC7216520

[34] Lüsebrink E Binzenhöfer L Kellnar A Müller C Scherer C Schrage B Venting during venoarterial extracorporeal membrane oxygenation. Clin Res Cardiol. (2023) 112(4):464–505. 10.1007/s00392-022-02069-0 35986750 PMC10050067

[35] Saeed S Vegsundvåg J. Usefulness of stress echocardiography in assessment of dynamic left ventricular obstructions: case series and review of the literature. Cardiology. (2021) 146(4):441–50. 10.1159/000516188 34004597

[36] Moura B Aimo A Al-Mohammad A Keramida K Ben Gal T Dorbala S Diagnosis and management of patients with left ventricular hypertrophy: role of multimodality cardiac imaging. A scientific statement of the heart failure association of the European society of cardiology. Eur J Heart Fail. (2023) 25(9):1493–506. 10.1002/ejhf.2997 37581253

[37] Tadic M Sala C Carugo S Mancia G Grassi G Cuspidi C. Myocardial strain and left ventricular geometry: a meta-analysis of echocardiographic studies in systemic hypertension. J Hypertens. (2021) 39(11):2297–306. 10.1097/HJH.0000000000002911 34128494

[38] Zhou Y Zhao CM Shen ZY Zhao X Zhou BY. Mitral early-diastolic inflow peak velocity (E)-to-left atrial strain ratio as a novel index for predicting elevated left ventricular filling pressures in patients with preserved left ventricular ejection fraction. Cardiovasc Ultrasound. (2021) 19(1):17. 10.1186/s12947-021-00248-z 33894780 PMC8070277

[39] Sato K Heinsar S Chan J Farah SM Wildi K Obonyo NG A novel echocardiographic parameter considering left ventricular afterload during V-A ECMO support. Eur J Clin Investig. (2024) 54(10):e14263. 10.1111/eci.14263 38849326

[40] Zhao Q Zhang Y Qiao Q Deng A. Prognostic factors of patients undergoing percutaneous coronary intervention supported by extracorporeal membrane oxygenation. J Pract Med. (2024) 40(2):219–24. 10.3969/j.issn.1006-5725.2024.02.016

[41] Jardin F Vieillard-Baron A. Right ventricular function and positive pressure ventilation in clinical practice: from hemodynamic subsets to respirator settings. Intensive Care Med. (2003) 29(9):1426–34. 10.1007/s00134-003-1873-1 12910335

