# Supplementary material for: Early left heart decompression protects the lungs in a canine model of acute left heart failure being treated with venoarterial extracorporeal membrane oxygenation
Source: Front Cardiovasc Med. 2025 Sep 24;12:1545903. doi: 10.3389/fcvm.2025.1545903 (PMC12504222; doi:10.3389/fcvm.2025.1545903)
Supplement: Supplementary file 1 [file Image1.pdf]

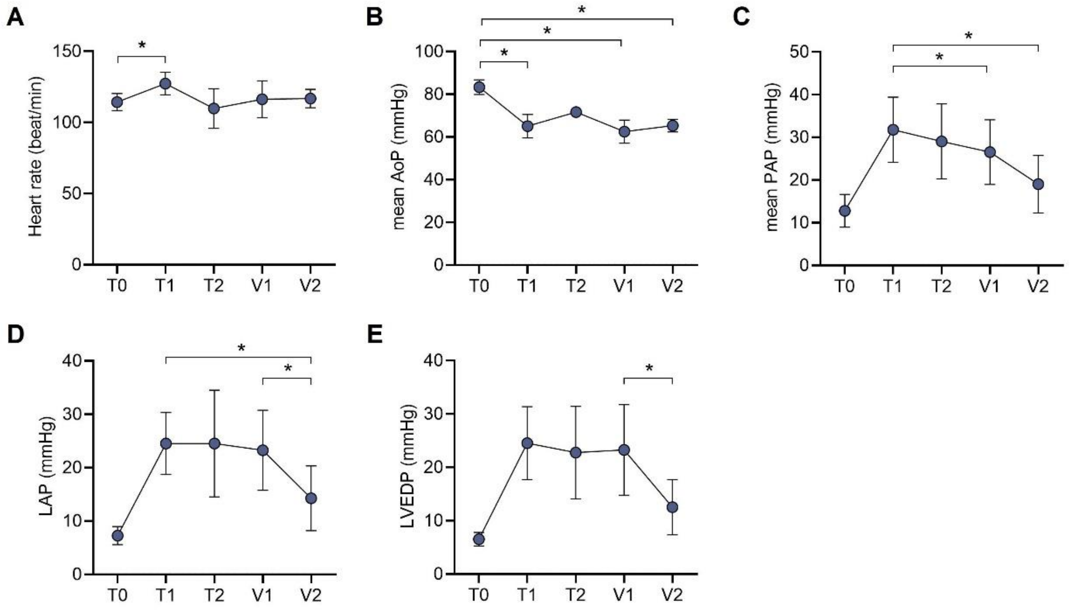

Supplementary Figure 1. The changes of hemodynamic indexes in dogs at different time. The changes of HR **(A)**, mean AoP **(B)**, mean PAP **(C)**, LAP **(D)**, LVEDP **(E)** of dogs at different time.

Data were present as mean ± SD, and were compared by one-way ANOVA. *，$P < 0.05$.
